# Balloon pulmonary angioplasty in patients with chronic thromboembolic pulmonary hypertension: short- and long-term results from a cohort in Brazil

**DOI:** 10.36416/1806-3756/e20240147

**Published:** 2024-12-17

**Authors:** Fabio Solano de Freitas Souza, Marcelo Gottschald Ferreira, Iury Andrade Melo, Marta Ferreira Leite de Sá, Camila Melo Coelho Loureiro, Rosalvo Abreu, Paulo Henrique Alves de Carvalho, Mateus dos Santos Viana, Valdemar Oliveira, Luiz Eduardo Fonteles Ritt

**Affiliations:** 1. Unidade de Intervenção Cardiovascular, Hospital Universitário Professor Edgard Santos, Universidade Federal da Bahia, Salvador (BA) Brasil.; 2. Instituto D’Or de Pesquisa e Ensino - IDOR - Hospital Cárdio-Pulmonar, Rede D’Or, Salvador (BA) Brasil.; 3. Clínica de Cirurgia Torácica - Cirtorax - Salvador (BA) Brasil.; 4. Centro de Referência de Hipertensão Pulmonar, Hospital Especializado Octávio Mangabeira, Salvador (BA) Brasil.; 5. Serviço de Pneumologia, Hospital Santa Izabel, Santa Casa da Misericórdia, Salvador (BA) Brasil.; 6. Serviço de Anestesiologia, Hospital Universitário Professor Edgard Santos, Universidade Federal da Bahia, Salvador (BA) Brasil.; 7. Escola Bahiana de Medicina e Saúde Publica, Salvador (BA) Brasil

**Keywords:** Hypertension, pulmonary, Pulmonary embolism, Angioplasty, balloon

## Abstract

**Objective:**

A significant number of patients with chronic thromboembolic pulmonary hypertension (CTEPH) are not eligible for pulmonary endarterectomy and may be treated with balloon pulmonary angioplasty (BPA). Although BPA programs have recently been developed in Brazil, no results have yet been published. The objective of this study was to assess the clinical and hemodynamic progression of the first patients treated with BPA at our center.

**Methods:**

This was an observational study of 23 patients with CTEPH enrolled in the BPA program of a specialized center in Brazil between 2015 and 2020.

**Results:**

After a mean of 5.6 ± 1.3 sessions and 11 ± 2.8 treated segments/patient (at a mean of 6.7 ± 2.9 months post-BPA), there was a 26% decrease in mean pulmonary artery pressure (51 ± 11 vs. 38 ± 11 mmHg; p < 0.0001), a 43% decrease in pulmonary vascular resistance (10 ± 3.7 vs. 5.7 ± 3.3 WU; p < 0.0001), and a 22.5% increase in the cardiac index (2.38 ± 0.6 vs. 2.95 ± 0.6 L/min/m^2^; p < 0.0001). There was an increase in the six-minute walk distance and an improvement in functional class. Acute lung injury with clinical manifestations was observed after 7% of the BPA sessions. None of the patients required intubation. During a mean outpatient follow-up period of 38 ± 22 months, two patients were referred for additional BPA sessions due to clinical worsening and new hospitalizations. Two deaths were recorded (due to CTEPH progression and gastrointestinal bleeding, respectively).

**Conclusions:**

Among this first group of patients treated with BPA in Brazil, there was significant short- and long-term clinical improvement, together with a low frequency of complications.

## INTRODUCTION

Chronic thromboembolic pulmonary hypertension (CTEPH) develops from the restriction of flow in pulmonary arterial branches by organized thrombi (remnants of previous pulmonary embolisms), leading to progressive increases in pulmonary vascular resistance (PVR) and pulmonary arterial pressure, as well as microvascular remodeling and right ventricular dysfunction.^1^ ^,^ ^2^ Patients typically present with dyspnea and reduced exercise capacity and may progress to right heart failure and death.^1^ ^,^ ^2^ The five-year survival rate is estimated to be 10% for patients who have a mean pulmonary artery pressure (mPAP) exceeding 50 mmHg and do not receive therapeutic interventions.^3^

Pulmonary endarterectomy (PEA), a potentially curative surgery, is the preferred therapy for eligible cases of CTEPH.^2^ ^,^ ^4^ ^-^ ^6^ However, approximately one-third of patients with CTEPH who are evaluated at expert centers are deemed to have inoperable disease due to severe comorbidities or involvement of distal arterial branches that are inaccessible to the surgeon or disproportionate to the increase in PVR.^7^ In these cases or when pulmonary hypertension (PH) persists post-PEA, pharmacological therapy has a limited effect on hemodynamic parameters, with no evidence of an impact on mortality.^4^ ^,^ ^6^ ^,^ ^8^ ^-^ ^11^

Balloon pulmonary angioplasty (BPA), an endovascular treatment, has the potential to significantly improve hemodynamics and functional capacity, representing an alternative for patients with inoperable CTEPH.^4^ ^,^ ^6^ ^,^ ^7^ ^,^ ^11^ ^-^ ^23^ Since the initial publications over two decades ago, the BPA technique has undergone numerous refinements, enhancing its safety and contributing to a global increase in the number of centers offering this treatment.^11^ ^,^ ^14^ ^-^ ^31^ Currently, BPA is mentioned in specific guidelines as an alternative for selected cases of inoperable CTEPH.^4^ ^,^ ^6^ In Brazil, no results of the use of this therapeutic approach have yet been published. In this study, we describe and analyze the short- and long-term outcomes of the first patients who underwent BPA in our group.

## METHODS

In this observational study, symptomatic patients with CTEPH, deemed candidates for BPA and followed on an outpatient basis at the University Hospital Professor Edgard Santos (HUPES) between February of 2015 and August of 2020, were sequentially selected for inclusion. The diagnosis of CTEPH was established using imaging methods (scintigraphy or computed tomography pulmonary angiography), together with invasive pulmonary angiography and right heart catheterization (RHC) showing an mPAP > 25 mmHg with a pulmonary artery occlusion pressure ≤ 15 mmHg and a PVR > 3 WU, after 3 months of effective anticoagulation (the definition prevailing at that time).^32^

All patients were assessed by our multidisciplinary team consisting of pulmonologists, interventional cardiologists, and thoracic surgeons. Contraindications to PEA were determined on the basis of anatomical and technical criteria, such as predominantly distal disease involvement, or on the basis of high surgical risk attributed to the presence of comorbidities or previous surgery. Patients with potentially operable CTEPH who did not have access to PEA due to difficulties related to the public health care system, or who refused to undergo surgery for personal reasons, were also assessed for BPA eligibility. The technical feasibility of performing BPA was determined by the interventional cardiologists on the team.

We included patients who completed treatment with BPA, underwent follow-up RHC 3-12 months thereafter, and were followed as outpatients at the HUPES for more than 12 months after the last angioplasty session. Patients who did not complete the treatment were excluded, as were those who were followed as outpatients at another facility.

Data were collected during outpatient medical consultations, as well as by reviewing medical records and procedure reports. Data collection was retrospective for the first seven patients and prospective for the remainder. This study was approved by the Research Ethics Committee of the HUPES, and all participating patients gave written informed consent.

### BPA procedure

Each BPA was performed in multiple sessions, in a staged manner, with intervals of more than one month between sessions. When possible, the first two BPA sessions were scheduled within 7 days of each other. The therapeutic target was the maximal reduction in mPAP until further pulmonary branch angioplasties were no longer possible, because of technical aspects or patient risk, or until there were no further complaints of dyspnea.

The procedure was conducted via femoral or internal jugular vein puncture under local anesthesia and mild sedation, with access to the pulmonary circulation via RHC. Unfractionated heparin at a dose of 5,000 IU was administered intravenously at the start of the procedure. A long (65-90 cm) 7-Fr sheath-Flexor Raabe (Cook Medical, Bloomington, IN, USA) or Destination (Terumo, Tokyo, Japan)-was inserted up to the pulmonary artery, after which a 6-Fr guiding catheter (Launcher Multipurpose or Launcher Judkins Right; Medtronic, Minneapolis, MN, USA) was inserted into the sheath. After the guiding catheter had been positioned in the target vessels, angioplasty was performed by advancing a 0.014” guide wire and inflating semi-compliant balloons in arterial segments with obstructive lesions and restricted flow, without the need for stent implantation. The duration of each session was generally limited to a fluoroscopy time of 60 min or a contrast volume of 300 mL, whichever came first.

To reduce the risk of acute pulmonary injury and vascular complications, balloons with a diameter of 2.0 mm or less than 50% of the reference vessel diameter were used in the initial sessions. In subsequent sessions, balloons close to the diameter of the treated vessels were used. All patients were observed in the intensive care unit for 24-48 h after each angioplasty session. Laboratory monitoring of renal function was conducted during hospitalization and after discharge.

In the initial patients treated, oral anticoagulation with coumarins was suspended 3-4 days before each BPA session. From 2019 onward, that suspension was requested only when the international normalized ratio (INR) was greater than 3.0. Direct oral anticoagulants were suspended the day before each session. In the absence of pulmonary or puncture site bleeding, oral anticoagulation was reintroduced within the first 12-24 h after each session. In cases of coumarin use, subcutaneous enoxaparin was used until an INR of 2.0 was achieved. All patients were discharged only after adjustment of oral anticoagulation.

### BPA outcomes

Hemodynamic, biochemical, and six-minute walk test (6MWT) data were obtained at baseline and at 3-12 months after the last BPA session. This follow-up interval was chosen to allow sufficient time for hemodynamic and clinical stabilization after the last BPA session, as well as to enable patients to undergo RHC and comprehensive clinical evaluations within the first year post-BPA. Medium- and long-term clinical follow-up data were obtained up to the last recorded medical contact. The patients were categorized by New York Heart Association functional class (NYHA-FC). Before and 3 months after treatment, quality of life was measured with the Minnesota Living with Heart Failure (MLHF) questionnaire.^33^

Procedural data, such as the number of BPA sessions, number of pulmonary segments addressed, and number of obstructive lesions treated, were obtained. Intravascular obstructive lesions were described according to an angiographic classification proposed in a previous study.^30^ Complications occurring during the procedures, such as vascular perforation, dissection, or both, and during in-hospital evolution, such as acute pulmonary injury and contrast-induced nephropathy, were identified.

### Statistical analysis

A descriptive analysis of the data was conducted. The main variables studied to evaluate the potential effectiveness of the treatment were short-term mPAP and PVR, and, from a functional standpoint, short- and long-term NYHA-FC. Acute lung injury with clinical manifestations was the main variable used in order to assess the safety of the treatment.

Continuous numerical variables are expressed as mean and standard deviation or as median and interquartile range, depending on their distribution. Categorical variables are expressed as absolute values and percentages. Comparative analyses between baseline and post-last session hemodynamic, clinical, and laboratory data were performed by using t-tests for paired samples with normal distribution, the Wilcoxon test for paired samples with non-normal distribution, or McNemar’s test for categorical variables. Values of p < 0.05 were considered statistically significant. Data analysis was performed with GraphPad Prism, version 10.2.2 for macOS (GraphPad Software, San Diego, CA, USA), www.graphpad.com.

## RESULTS

**Figure 1:**
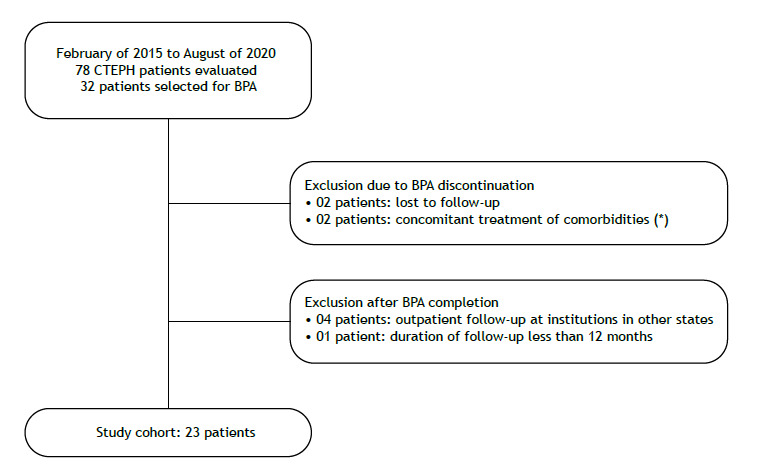
Flow chart of the patient selection process. *One patient had congenital isolated stenoses in the right arterial interlobar pulmonary branch, concurrent with CTEPH lesions in the segmental vessels of the left lung. After significant hemodynamic and clinical improvements following stent implantation in the right interlobar branch, this patient was excluded from the study cohort to prevent confusion in interpreting the effects of BPA. The other patient had a significant psychiatric disorder that complicated the performance of BPA sessions and the interpretation of clinical outcomes. After three BPA sessions, we decided to prioritize treatment of the psychiatric condition. BPA: balloon pulmonary angioplasty; and CTEPH, chronic thromboembolic pulmonary hypertension.

Between February of 2015 and August of 2020, 23 of 32 patients selected for BPA were included in the analysis (Figure 1; Table 1). The reasons for BPA indication were technically inoperable distal disease in 15 patients (65%), high-risk comorbidities for PEA in 2 (9%), lack of access to PEA in 5 (22%), and refusal to undergo surgery in 1 (4%).

**Table 1:** Baseline characteristics of the study cohort.

Baseline characteristic	(N = 23)
Age (years), mean ± SD	50 ± 14
Female sex, n (%)	20 (87)
BMI (kg/m^2^), mean ± SD	27.3 ± 4.5
Previous VTE, n (%)	12 (52)
Thrombophilia, n (%)	4 (17)
Hypertension, n (%)	12 (52)
Previous PEA, n (%)	1 (4)
Symptom duration (months), median (IQR)	36 (24-54)
Time from diagnosis to BPA, median (IQR)	14 (9-28)
NYHA-FC II/III/IV, n/n/n	0/11/12
Patients on oxygen therapy, n (%)	7 (30)
Medications in use, n (%)	
Riociguat	5 (22)
Sildenafil	15 (65)
Bosentan	8 (35)
No vasodilator	1 (4)
Furosemide	18 (78)
mPAP (mmHg), mean ± SD	51 ± 10.7
PVR (WU), mean ± SD	10 ± 3.7
RAP (mmHg), mean ± SD	13 ± 5.3
CI (L/min/m^2^), mean ± SD	2.4 ± 0.6
SvO_2_ (%), mean ± SD	57.7 ± 9
Hemoglobin (g/dL), mean ± SD	14 ± 1.9
Creatinine (mg/dL), mean ± SD	0.9 ± 0.19
Urea (mg/dL), mean ± SD	33 ± 7.8

BPA: balloon pulmonary angioplasty; VTE: venous thromboembolism; PEA: pulmonary endarterectomy; NYHA-FC: New York Heart Association functional class; mPAP: mean pulmonary artery pressure; PVR: pulmonary vascular resistance; RAP: right atrial pressure; CI: cardiac index; and SvO_2_: mixed venous oxygen saturation.

The majority of the patients were female. In the sample as a whole, the mean age was 50 ± 13.8 years and the median symptom duration prior to the first BPA session was 36 months (IQR, 24-54 months). All patients exhibited exertional dyspnea and NYHA-FC III or IV, despite pharmacological treatment. Nearly all of the patients were under continuous treatment with a pulmonary vasodilator at stable doses, with a median treatment duration of 6 months (IQR, 3-8 months). Five of the 6 patients with potentially operable CTEPH were treated with pulmonary vasodilators only after the decision to undergo BPA had been made. Only 5 patients were taking riociguat (a soluble guanylate cyclase stimulator) before BPA.

The conclusion of BPA treatment was based on the absence of any remaining vessels technically suitable or anatomically relevant for intervention in 14 cases (61%), patient choice following perceived sufficient clinical improvement in 5 cases (21%), lack of clinical improvement in 2 cases (9%), and an unfavorable risk-benefit ratio in 2 cases (9%).

**Figure 2:**
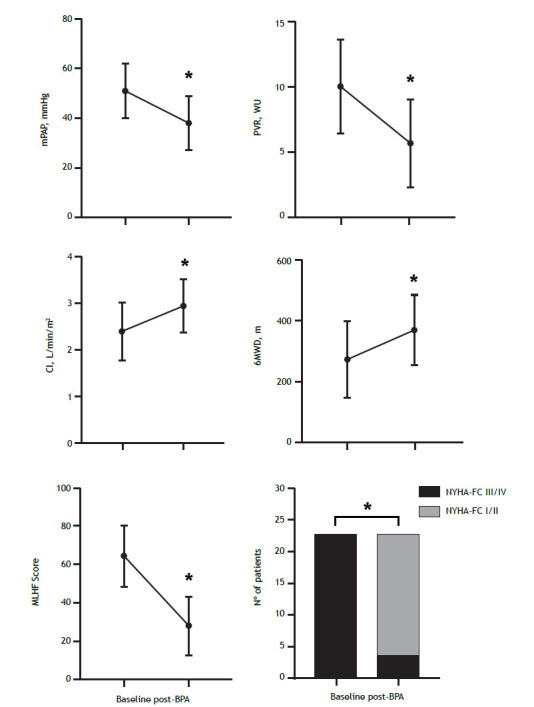
Effects of balloon pulmonary angioplasty (BPA) on clinical and hemodynamic parameters. BPA: balloon pulmonary angioplasty; mPAP: mean pulmonary artery pressure; PVR: pulmonary vascular resistance; CI: cardiac index; 6MWD: 6-minute walk distance; NYHA-FC: New York Heart Association functional class; MLHF: Minnesota Living with Heart Failure (questionnaire). *p < 0.0001.

Patients underwent a mean of 5.6 ± 1.3 sessions of BPA over a mean time of 12.5 ± 7.0 months (Table 2). The mean of pulmonary segments addressed per patient was 11 ± 2.7. Follow-up RHC was performed for hemodynamic reevaluation at a mean of 6.7 ± 3.0 months after the last angioplasty session. Comparing the values obtained at the end of treatment with those obtained at baseline, we observed significant improvements in hemodynamic parameters, including a 26% reduction in mPAP (51 ± 11 mmHg vs. 38 ± 11 mmHg; p < 0.0001), a 43% decrease in PVR (10 ± 3.7 mmHg vs. 5.7 ± 3.4 mmHg; p < 0.0001), and a 22.5% increase in the cardiac index (2.38 ± 0.6 L/min/m^2^ vs. 2.95 ± 0.6 L/min/m^2^; p < 0.0001). In addition, an mPAP lower than 25 mmHg was achieved in 4 patients. Within the first 6 months after the last session, there was also a reduction in the median plasma level of B-type natriuretic peptide (256 pg/mL [IQR, 130-180 pg/mL] vs. 46 pg/mL [IQR, 19-130 pg/mL]; p < 0.0001), an increase in the mean 6-minute walk distance (273 ± 127 m vs. 370 ± 114 m; p < 0.0001), and symptom improvement with reductions in NYHA-FC and in home oxygen use (Table 3; Figure 2). The 6MWT was performed a median of 4 months (IQR, 3-7 months) after the last BPA session. The quality of life of the patients, as determined by the mean MLHF score, improved significantly following the angioplasties (64.5 ± 16 vs. 27.7 ± 15.5; p < 0.0001). In a patient who had received sildenafil monotherapy prior to BPA, the pulmonary vasodilator treatment was withdrawn. It was also possible to discontinue furosemide in 8 of the 18 patients who were using it before BPA (p = 0.008).

**Table 2:** Procedural data and complications.

Variable	Value
Treatment duration (months), mean ± SD	12.5 ± 6.8
BPA sessions, n	128
Number of BPA sessions/patient, mean ± SD	5.6 ± 1.3
Fluoroscopy time (min/session), mean ± SD	60 ± 17.3
Contrast medium volume (mL/session), mean ± SD	295 ± 65
Treated lung segments, n	253
Treated lung segments (n/patient), mean ± SD	11 ± 2.7
Untreated lung segments (n/patient), mean ± SD	3 ± 1.8
Treated obstructive lesions, n	422
Lesion type, n (% of all lesions)	
A	48 (11.4)
B	252 (59.7)
C	85 (20.0)
D	28 (6.6)
E	9 (2.1)
Procedure-related complications, n (% of all sessions)	
Acute lung injury	9 (7)
Hemoptysis	9 (7)
Mechanical ventilation	0
NPPV	4 (3.1)
Arterial perforation	6 (4.7)
Arterial dissection	20 (15.6)
Acute kidney injury	2 (1.6)

BPA, balloon pulmonary angioplasty; and NPPV, Non-invasive positive pressure ventilation.

**Table 3:** Effects of balloon pulmonary angioplasty on hemodynamic, laboratory and clinical parameters.

Parameter	Baseline	Final	Variation		P value
			Absolute	Relative	
sPAP (mmHg), mean ± SD	91 ± 17	69 ± 19.4	− 22	− 24%	< 0.0001
mPAP (mmHg), mean ± SD	51 ± 10.7	38 ± 11	− 13.2	− 25.9%	< 0.0001
PVR (WU), mean ± SD	10 ± 3.7	5.7 ± 3,4	− 4.3	− 43.0%	< 0.0001
CI (L/min/m^2^), mean ± SD	2.38 ± 0.6	2.95 ± 0.6	+ 0.46	+ 22.5%	< 0.0001
RAP (mmHg), mean ± SD	13 ± 5.3	8.8 ± 4.9	− 4.2	− 32%	0.0012
HR (bpm), mean ± SD	85 ± 11.4	74 ± 12	− 10.7	− 12.6%	< 0.0001
SvO_2_ (%), mean ± SD	57.7 ± 9	67.4 ± 6	+ 9.7	+ 16.8%	< 0.0001
SaO_2_ (%), mean ± SD	92 ± 4.2	95.5 ± 3.2	+ 3.5	+ 3.8%	0.0002
BNP (pg/mL), median (IQR)	256 (130-380)	46 (19-130)	− 168	− 56.8%	< 0.0001
6MWD (m), mean ± SD	273 ± 127	370 ± 113.8	+ 97	+ 35.5%	< 0.0001
SpO_2_ at peak exercise, mean ± SD	85.3 ± 8	89.6 ± 4.5	+ 4.3	+ 5.0%	0.0068
Hemoglobin (g/dL), mean ± SD	14 ± 1.9	13.3 ± 1.3	− 0.7	− 5%	0.09
Creatinine (mg/dL), mean ± SD	0.9 ± 0.19	0.9 ± 0.2	− 0.02	0%	0.5
Oxygen therapy, n	7	3	− 4	− 57%	0.13

sPAP: systolic pulmonary artery pressure; mPAP: mean pulmonary artery pressure; PVR: pulmonary vascular resistance; RAP: right atrial pressure; CI: cardiac index; SvO_2_: mixed venous oxygen saturation; BNP: plasma B-type natriuretic peptide; and 6MWD: six-minute walk distance.

**Figure 3:**
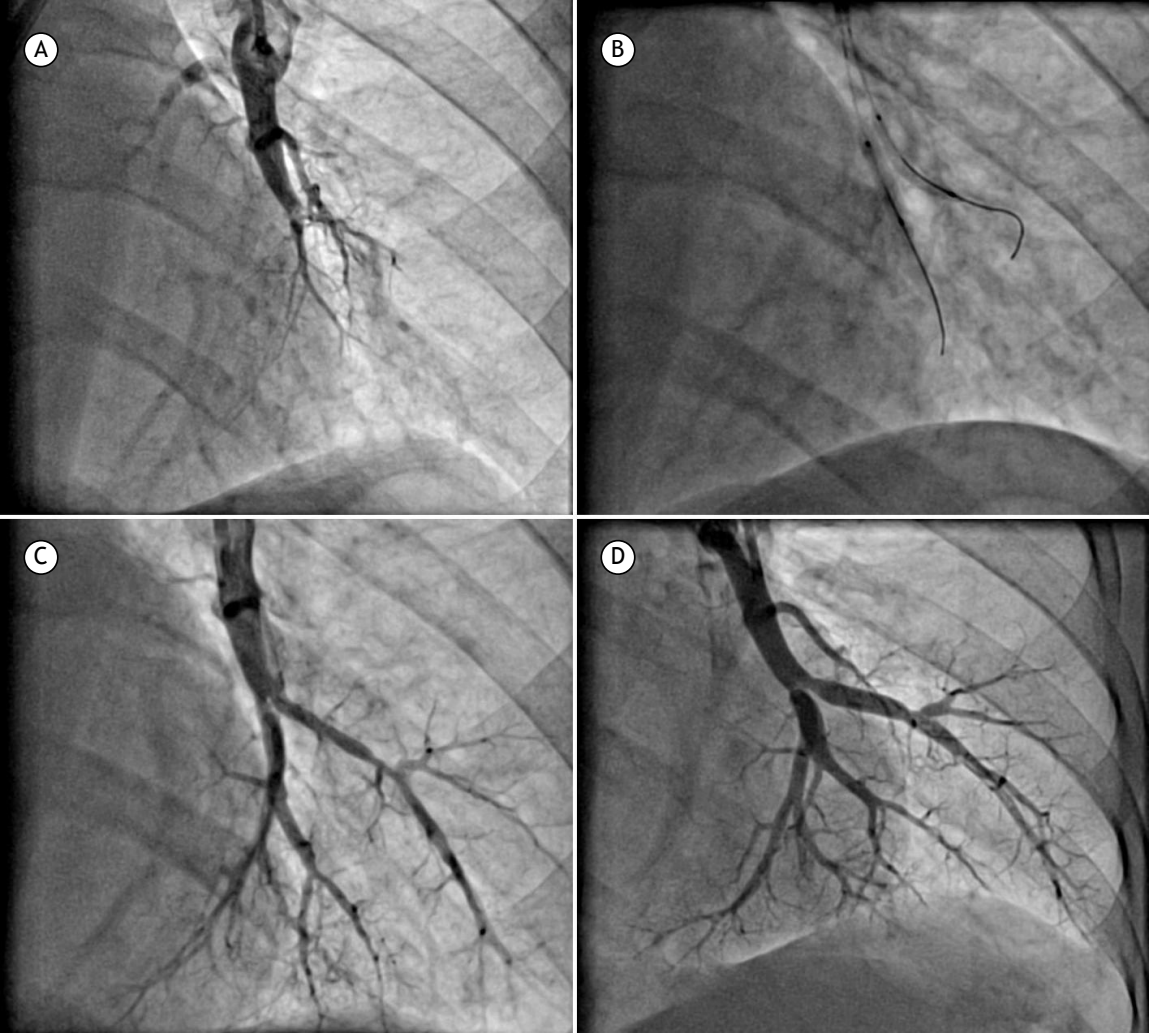
Balloon pulmonary angioplasty of arterial branches (A8 and A7) to anterior and medial segments of the left inferior lobe, depicted by selective pulmonary angiograms from A to D. A: Suboccluded branches (type C obstructive lesions) with distal narrowing and flow reduction. B: Balloon pulmonary angioplasty. C: Immediate result. D: Selective angiogram after 6 months, demonstrating vascular patency and positive remodeling.

Obstructive lesions were located in the lower lobes in 55% of the interventions, in the upper lobes in 20%, in the middle lobe in 18%, and in the lingula in 8%. The majority (80%) of the obstructive lesions addressed were type B, corresponding to lesions appearing as “webs” or “slits” within vessels, or type C, corresponding to subocclusive vascular lesions (Table 2; Figure 3). Interventions in type D and E lesions were associated with vascular complications in 28% and 11%, respectively, whereas interventions in type A, B, and C lesions were associated with a lower rate of complications (2.0%, 4.4%, and 6.0%, respectively). Vascular dissections and perforations were observed in 15.6% and 4.7% of the 128 sessions, respectively, usually without immediate clinical repercussions. Only 2 vascular perforations (1.6%) resulted in hemoptysis during the procedure and were treated through prolonged proximal balloon inflation and reversal of anticoagulation, without the need for vascular embolization. Acute lung injury with clinical manifestations was observed after 9 (7%) of the 128 sessions. Clinical manifestations were characterized by dyspnea at rest, with or without hemoptysis, or a drop in SpO_2_ accompanied by pulmonary crackles upon auscultation of the thoracic regions in which the segments subjected to interventions were located, within the first 48 h post-BPA. Noninvasive positive pressure ventilation (NPPV) was required in 4 interventions (3%), and nasal oxygen supplementation was required in the others, all patients showing gradual improvement 3-5 days after BPA. Acute kidney injury was detected following 2 (1.6%) of the BPA sessions, with spontaneous reversal of creatinine to baseline values within a few days of follow-up.

The mean duration of the clinical outpatient follow-up period was 38 ± 22 months, during which time most of patients maintained the same NYHA-FC. Clinical worsening with hospital admissions was observed in 4 patients with a median time to hospitalization of 39 months (IQR, 13.8-64.0 months). Two of those patients were referred for another series of BPA sessions after angiographic reevaluation, to treat arterial branches not addressed in the initial series. There were two deaths unrelated to the pulmonary angioplasties: one in a 26-year-old patient, from progressive PH, at 7 months after BPA (considered a nonresponder to the interventional treatment); and the other in a 53-year-old patient, from hemorrhagic shock secondary to gastrointestinal bleeding associated with oral anticoagulation with warfarin, 44 months after the last BPA session.

## DISCUSSION

This observational study describes the short- and long-term effectiveness and safety of BPA as an alternative to PEA as a therapeutic approach for symptomatic patients with CTEPH, conducted by a PH referral medical group in Brazil. Ours represent the first published BPA results for Brazil.

In this study, pulmonary angioplasties were indicated after careful assessment by a specialized multidisciplinary team as part of the proposed treatment protocol for patients with CTEPH, reflecting the medical practice of the team during the study period. The hemodynamic criteria used in order to define PH were those in effect at the time of patient selection.^32^ Thus, in accordance with the latest diagnostic criteria,^34^ patients with CTEPH who had an mPAP of 20-25 mmHg were not included.

Follow-up RHCs performed after BPA demonstrated significant hemodynamic improvement, including reductions in pulmonary arterial and right chamber pressures, decreased PVR, and increased cardiac index associated with a reduction in resting heart rate, indicating increased ventricular systolic volume. The respective 26% and 43% decreases in mPAP and PVR (the primary hemodynamic variables investigated) were not as pronounced in our patient sample as in those evaluated in studies conducted in Japan, in which reductions ranging from approximately 40% to approximately 60% were reported.^14^ ^,^ ^15^ ^,^ ^17^ ^-^ ^22^ The more modest hemodynamic impact observed in our study could be attributed to a few factors. The first is the learning curve effect on the interventional cardiologists who performed the angioplasties, given that these were the first patients treated by our group. In addition, the mean baseline mPAP in our sample was higher than that reported in most previous studies of BPA. Furthermore, the long duration of symptoms prior to BPA suggested that our patients had advanced CTEPH and were therefore at a higher risk of advanced microvascular disease, which might diminish the response to endovascular treatment. Another potential factor is the selection criteria for BPA and PEA used by our group. At centers in Japan, the majority of patients with CTEPH are referred for BPA, whereas the inverse occurs at centers in Europe and North America.^7^ Our criteria seem more aligned with European and North American standards, although we are partly constrained to refer patients for surgery due to limitations related to the public health care system in Brazil, leading to some patients with potentially operable disease undergoing BPA. When comparing the hemodynamic results obtained at our center with those obtained at centers in Europe, we observed similar improvements in mPAP, PVR, and the cardiac index.^23^ ^-^ ^25^ Moreover, we cannot disregard possible genetic differences among the populations of Japan, Europe, and Brazil that may influence disease characteristics and treatment responses.

The frequency of adverse events in our study was low compared with that reported in previous studies, likely due to our less aggressive interventional approach, which might also have influenced the extent of hemodynamic improvement observed. Vascular dissections and perforations were not associated with clinical manifestations in most cases. Among the 128 sessions performed, perforations that required immediate endovascular intervention occurred in only 2. Acute lung injury with clinical manifestations was observed after 9 (7%) of the sessions, affecting 6 (26%) of our patients, and NPPV was required in 4 events involving 3 of those patients. Mechanical ventilation was not required for any of the patients in our sample, and there were no in-hospital deaths.

Clinically, BPA led to significant improvement in NYHA-FC and in functional capacity assessed by the 6MWT. Discontinuation of diuretics and home oxygen was possible in a significant proportion of patients. The NYHA-FC remained unchanged in most patients throughout the extensive clinical follow-up period. New hospital admissions occurred in 3 patients due to worsening of symptoms related to CTEPH and in 1 patient due to worsening dyspnea associated with the progression of chronic mitral valve disease. Among the 3 patients with worsening CTEPH, 2 underwent a new series of BPA sessions after reevaluation by interventional cardiologists, because these were among the first patients treated by our group, in whom obstructive lesions that were more complex had not initially been addressed because of technical limitations of the learning curve. After the new series, those patients reported further symptom improvement.

An improvement in quality of life was evidenced by the decrease in MLHF scores at 3 months after BPA. We used the Portuguese-language version of the MLHF questionnaire to evaluate the patients in our study, considering the similarity of symptoms and impact on quality of life in patients with right cardiac effects associated with CTEPH, compared with patients with left heart failure, for whom the MLHF questionnaire was originally created and validated.^33^ Therefore, its applicability in individuals with PH not associated with left heart disease, like those in the present study, has yet to be validated, and we should interpret the results as hypothesis-generating. In addition to all of the previously mentioned limitations, the present study also has the limitations inherent to a single-center observational study, including the lack of a control group.

In conclusion, the present study describes significant hemodynamic and clinical improvement, together with a low frequency of complications, in the first patients with CTEPH undergoing BPA by a specialized group in Brazil. The study population, which presented signs of advanced PH, impaired quality of life, and a guarded prognosis, showed good long-term clinical progression after completion of the interventional treatment. 
